# Supplementary material for: Ultra-Long-Term Delivery of Hydrophilic Drugs Using Injectable In Situ Cross-Linked Depots
Source: bioRxiv. 2024 Aug 28:2023.11.04.565631. Preprint. [Version 2] doi: 10.1101/2023.11.04.565631 (PMC11382995; doi:10.1101/2023.11.04.565631)
Supplement: Supplement 1 [file media-1.pdf]

Supplementary Information

Ultra-Long-Term Delivery of Hydrophilic Drugs Using Injectable *In Situ* Cross-Linked Depots

Sohyung Lee[1,2,3]†, Spencer Zhao[2,3]†, Weihua Jiang[4], Xinyang Chen[2,3], Lingyun Zhu[2,3], John Joseph[1,2,3], Eli Agus[2,3], Helna Baby Mary[2,3], Shumaim Barooj[2,3], Kai Slaughter[2,3], Krisco Cheung[2,3], James N Luo[1,5], Chetan Shukla[2,3], Jingjing Gao[1,2,3,6], Dongtak Lee[1,2,3], Biji Balakrishnan[7], Christopher Jiang[2,3], Amogh Gorantla[2,3], Sukyung Woo[4]*, Jeffrey M Karp[1,2,8,9,10]* and Nitin Joshi[1,2,3]*

[1] Harvard Medical School, Boston, MA, USA

[2] Center for Accelerated Medical Innovation, Department of Anesthesiology, Perioperative and Pain Medicine, Brigham and Women's Hospital, Boston, MA 02115, USA

[3] Center for Nanomedicine, Department of Anesthesiology, Perioperative and Pain Medicine, Brigham and Women's Hospital, Boston, MA, USA

[4] Department of Pharmaceutical Sciences, School of Pharmacy and Pharmaceutical Sciences, The State University of New York at Buffalo, Buffalo, NY 14215, USA

[5] Department of Surgery, Brigham and Women's Hospital, Boston, MA 02115, USA

[6] College of Engineering, University of Massachusetts Amherst, MA, USA

[7] Somaiya Centre for Integrated Science education and research, SKSC, Somaiya Vidyavihar University, Mumbai, 400077, India

[8] Harvard–Massachusetts Institute of Technology Division of Health Sciences and Technology, Massachusetts Institute of Technology, Cambridge, MA 02139, USA

[9] Broad Institute, Cambridge, MA 02142, USA.

[10] Harvard Stem Cell Institute, Cambridge, MA 02138, USA

†Equal contribution

*Corresponding authors: S.W. (skwoo@buffalo.edu) , J.M.K. (jmkarp@bwh.harvard.edu) and N.J. (njoshi@bwh.harvard.edu)

This PDF file includes:

Supplementary Figure 1 to 13

Supplementary Tables 1 to 6

SUPPLEMENTARY FIGURES

A

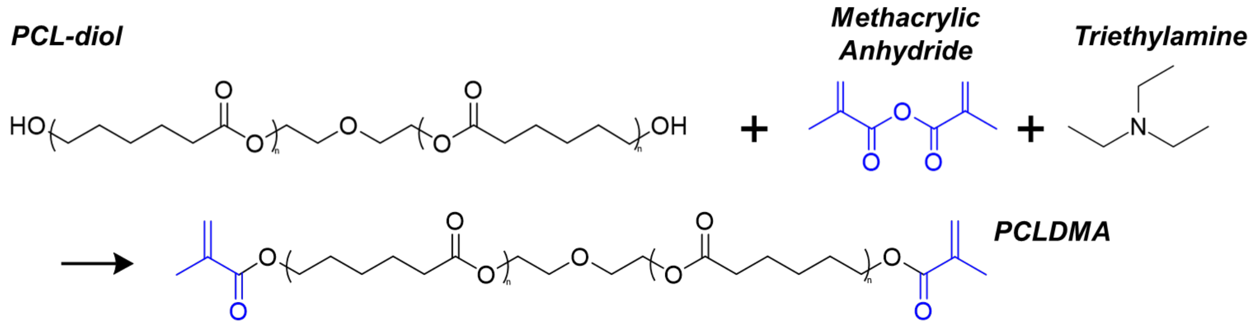

B

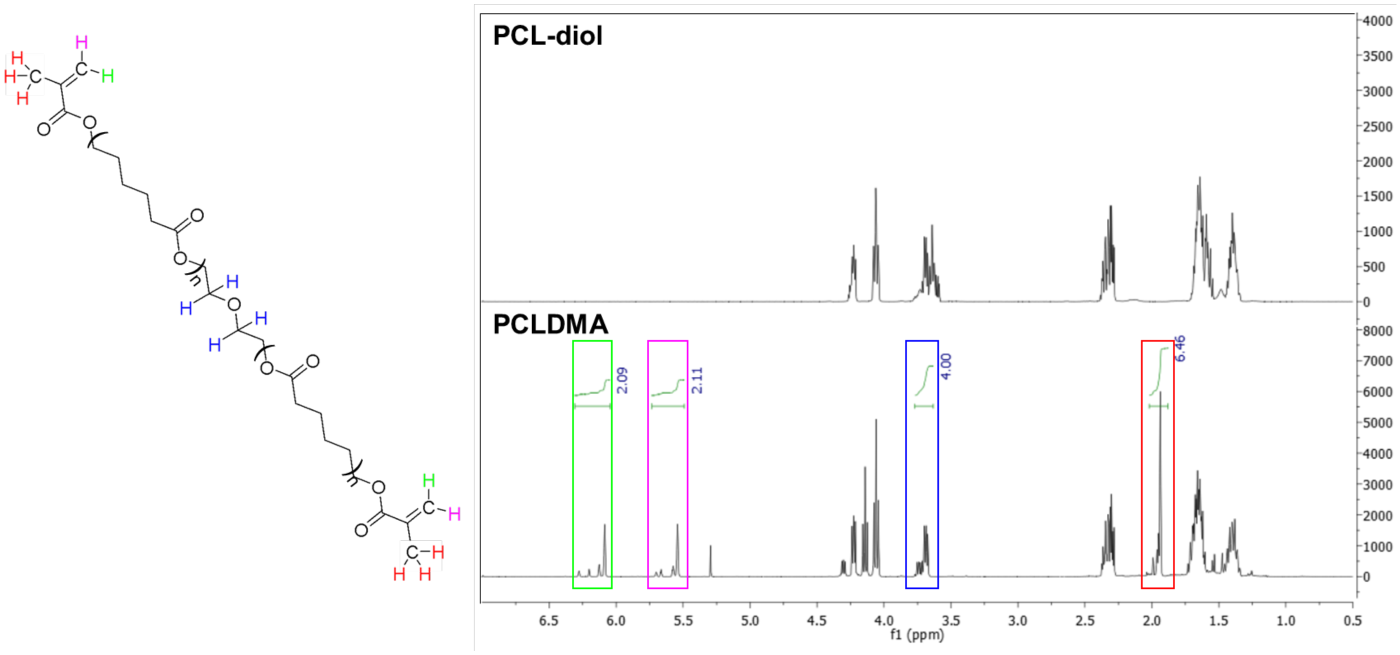

Figure S1. Synthesis and characterization of PCLDMA. A. Schematic showing methacrylation of hydroxyl-functionalized PCL to yield PCLDMA. **B.** ^{1}H-NMR spetrcum of of PCLDMA. The chemically equivalent protons are labeled with the same color and their NMR signals are marked with the same colored box for ease of understanding. The NMR integration of each signal corresponds to the expected ratio of each type of hydrogen in the PCLDMA molecule.

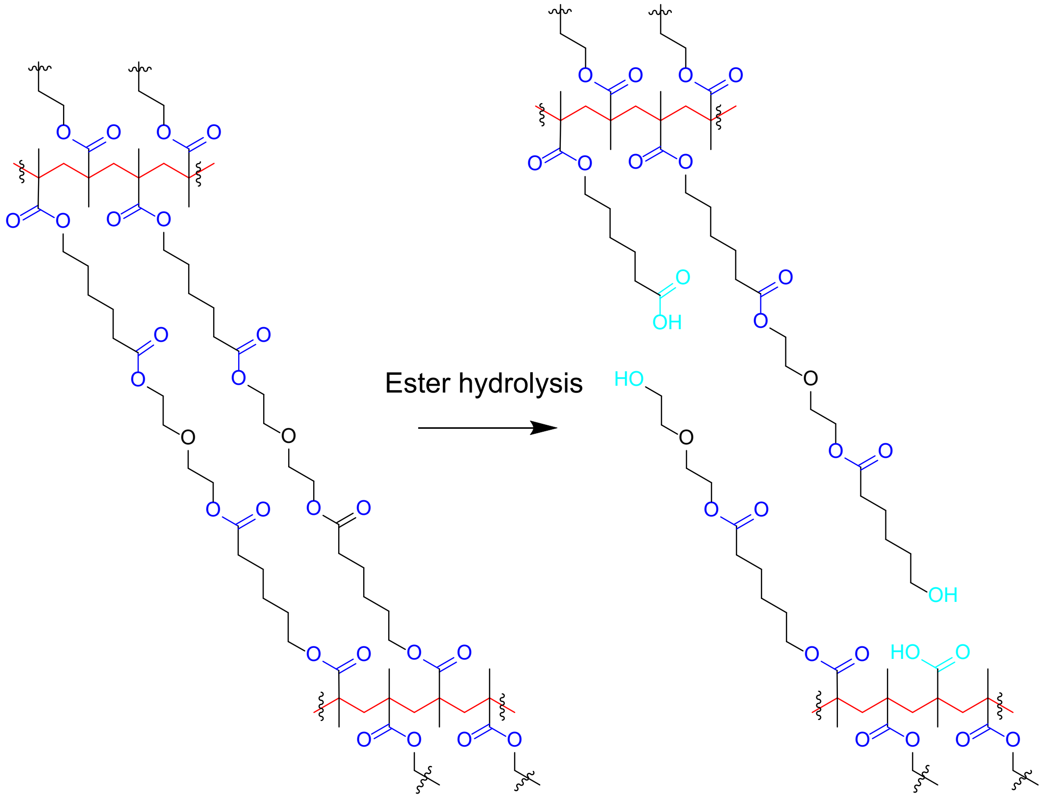

Figure S2. Schematic illustrating hydrolysis of polycaprolactone domains and subsequent collapse of ISCD. The ISCD consists of a cross-linked network of polymethacrylate chains (red) connected by ester bonds (dark blue). Hydrolysis of these ester bonds (light blue) disconnects the polymethacrylate chains, enabling the depot to degrade.

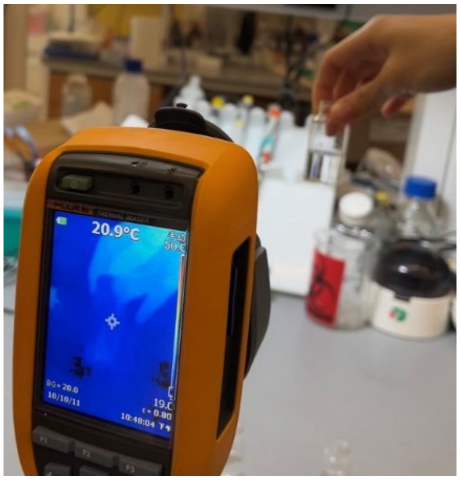

Figure S3. Infrared thermal imaging confirms that there is no noticeable heat generated during ISCD polymerization.

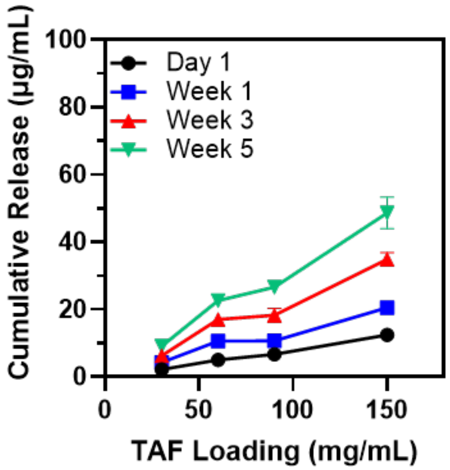

Figure S4. *In vitro* cumulative release of TAF from ISCD loaded with different concentrations of TAF and incubated in PBS (37°C). Data are presented as mean ± standard deviation (n=3, experiments performed at least twice).

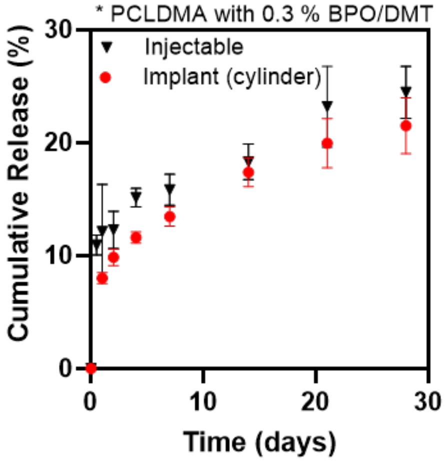

Figure S5. *In vitro* cumulative release of TAF from ISCD depots formed by injecting pre-polymer mixture into PBS (37°C) compared with TAF release from pre-formed implants with cylindrical shape. Data are presented as mean ± standard deviation (n=3, experiments performed at least twice).

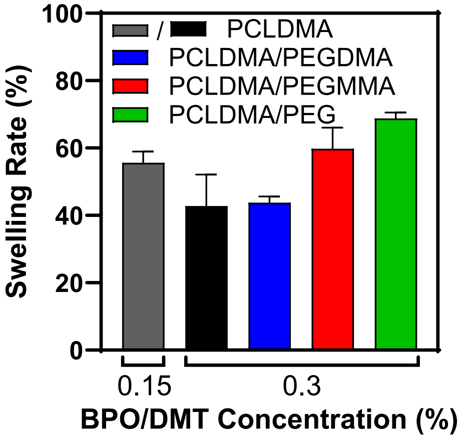

Figure S6. Swelling rate of different ISCD formulations studied in benzyl alcohol aftera week. Data are presented as mean ± standard deviation (n=3, experiments performed at least twice).

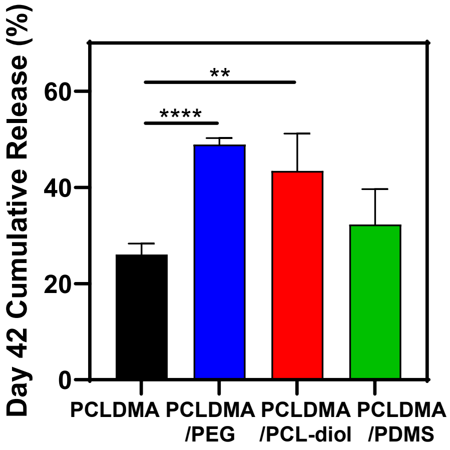

Figure S7. Cumulative release of TAF at day 42 post-incubation of different ISCD formulations in PBS (37°C). (**P<0.01, ****P<0.0001). Data are presented as mean ± standard deviation (n=3). The P-value was determined using one-way ANOVA with Tukey's post hoc analysis.

.

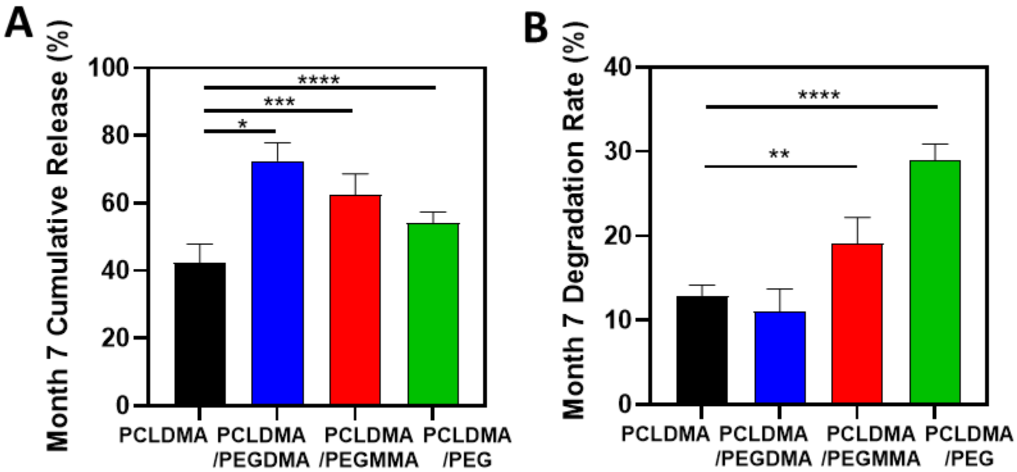

Figure S8. Impact of incorporating external polymer additives with varying degrees of methacrylation into ISCD on TAF release and depot degradation. A. Month 7 cumulative release, and B. month 7 degradation rate of unmodified ISCD and ISCD containing 25 wt% of PEGs with different degree of methacrylation, when incubated in PBS (37°C). (*$P<0.05$, **$P<0.01$, ***$P<0.001$, ****$P<0.0001$). Data are presented as mean ± standard deviation (n=3). The *P*-value was determined using one-way ANOVA with Tukey's post hoc analysis.

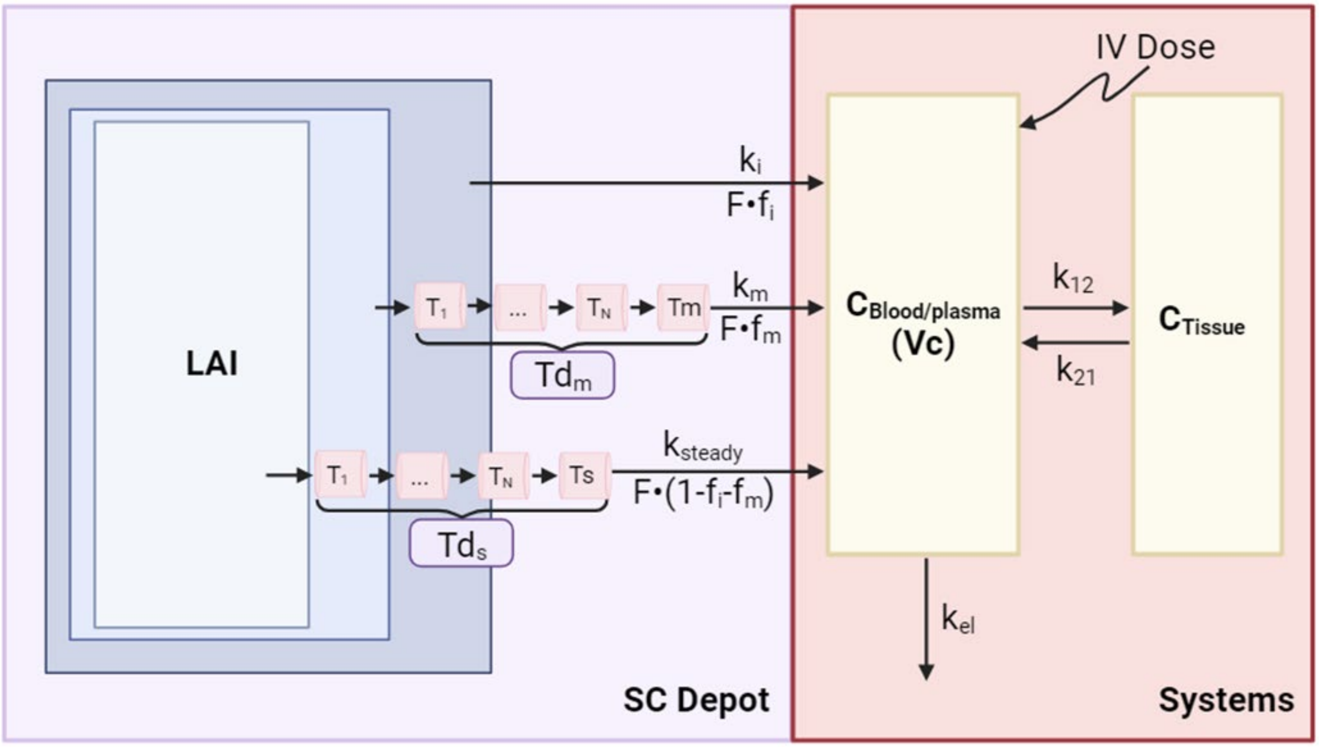

Figure S9. The scheme of the pharmacokinetic (PK) model for subcutaneous injection of ISCD. The disposition kinetics (referred to as Systems) of analytes is characterized by two compartments ($C_{Blood/Plasma}$ and C_{Tissue}), with first-order rate constants for elimination (k_{el}), distribution (k_{12}), and redistribution (k_{21}), and Vc for the central volume of distribution. At the SC implant site (referred to as SC Depot), the release/absorption model assumes three sequential release phases, delineated by first-order release rate constants (k_i, k_m, k_s). Initially, a fraction (f_i) of the ISCD implant is released (k_i), leading to the maximum concentration in the central compartment. Subsequently, drug release continues with an intermediate phase (k_m) for a fraction of the total released drug mass (f_m), followed by a sustained release phase (k_s) for the remaining drug amount (1- f_i- f_m). The time delays associated with the intermediate (Tdm) and sustained-release (Tds) phase are characterized by a gamma distribution function with shape (N) and rate parameter (Td). F represents the bioavailability of ISCD implants.

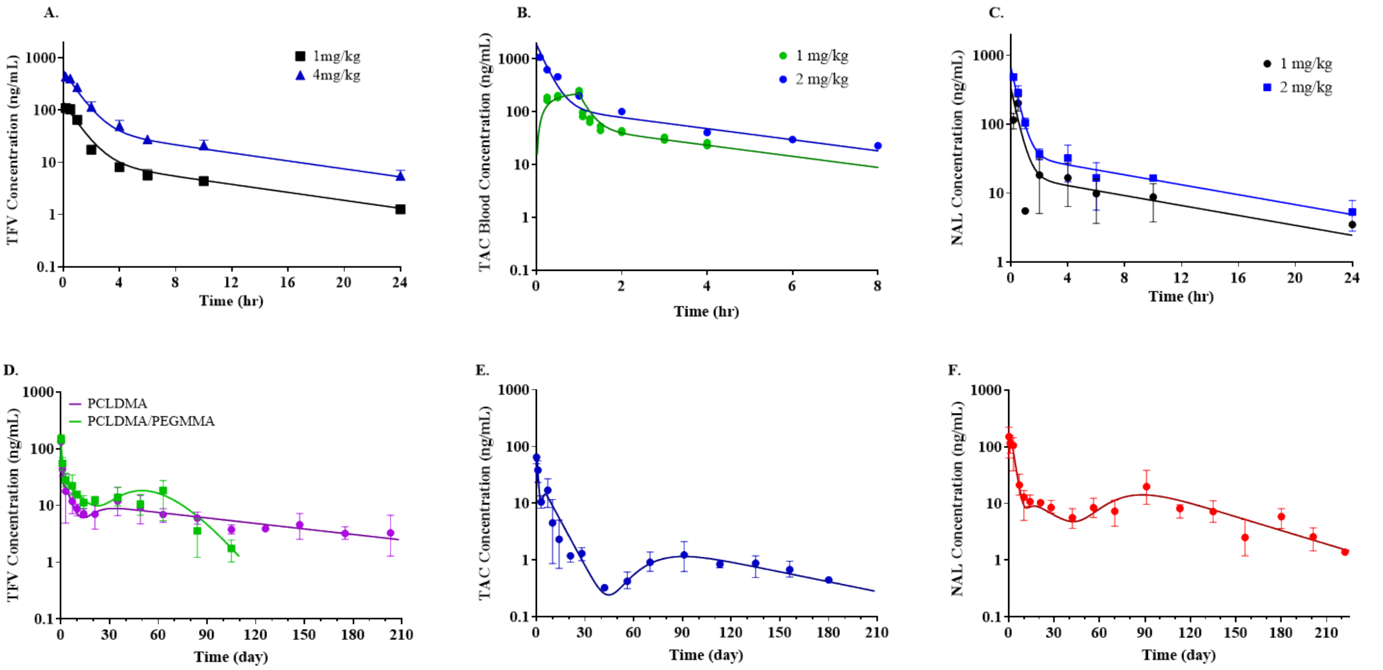

Figure S10. Time profiles of the blood/plasma concentration in rats after a single IV dose of **A.** TFV (1 & 4 mg/kg) **B.** TAC (1 & 2 mg/kg), and **C.** NAL (1 & 2 mg/kg). Time profiles of the blood/plasma concentration in rats after subcutaneous injection of ISCD containing **D.** TFV, **E.** TAC, and **F.** NAL. Data presented as symbols reflect the mean ± standard deviation of three technical repeats (n=3). Lines represent the model-predicted drug concentrations.

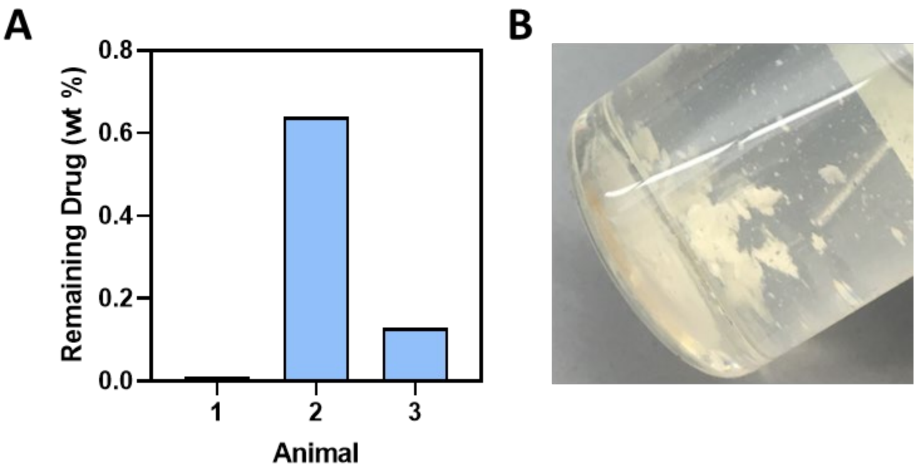

Figure S11 A. Pecentage of the initial amount TAF amount remaining in the explanted ISFI following the 2-month *in vivo* study in rats. B. ISFI explanted after the 2-month *in vivo* study is a fragmented solid. Data are presented as individual values for each animal.

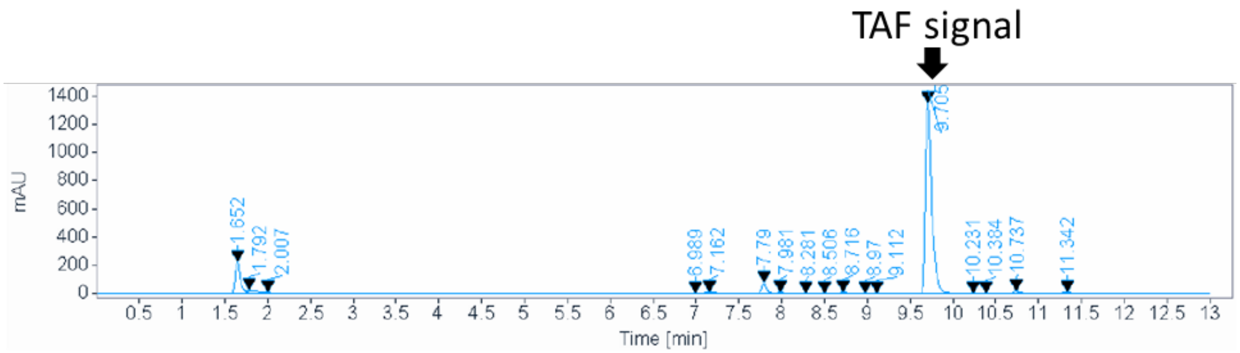

Figure S12. HPLC chromatograph of TAF-loaded ISCD explanted after a 7-month *in vivo* study.

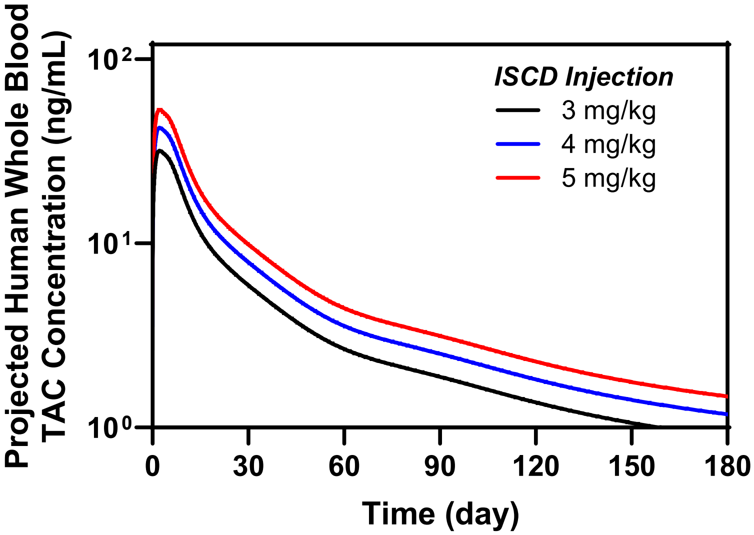

Figure S13. Convolution analysis-based prediction of human PK of a single subcutaneous dose of TAC-loaded ISCD (at different dosages) upto 6 months.

SUPPLEMENTARY TABLES

Table S1. Initial burst release against water solubility of therapeutics

Drug	Solubility (mg/ml)	Cumulative release after 24 h (%)
FTC	112	18.51
NAL	100	22.02
LAM	70	14.5
VAN	50	14.98
TAF	5.63	12.14
AMX	3	0.79
ABC	1.21	6.04
TAC	0.004	1.34

Table S2. The estimated PK parameters of disposition and release kinetics were obtained from the concentration-time profiles following IV administration and SC implants of ISCDs in rats.

Parameters	Definition	PCLDMA +TAF	PCLDMA/ PEGMMA +TAF	PCLDMA +TAC	PCLDMA +NAL
Disposition Kinetics					
**V_c (mL)**	Central compartment volume	1266 (9)[a]		306 (17)	923 (20)
**k_{el} (1/hr)**	Elimination rate constant	0.558 (8)		2.06 (16)	0.925 (17)
**k_{12} (1/hr)**	Transfer rate constant from central to peripheral compartment	0.372 (15)		2.08 (21)	1.22 (19)
**k_{21} (1/hr)**	Transfer rate constant from peripheral to central compartment	0.158 (12)		0.517 (21)	0.203 (17)
**$t_{1/2}$ (hr)**	Terminal elimination half-life	7.88		2.87	8.35
**CL (L/hr)**	Total systemic clearance	707		631	853
Release Kinetics					
**k_i (1/day)**	Initial release rate constant	2.47 (27)	2.52 (25)	0.962 (43)	0.372 (13)
**f_i**	Fraction of the released drug mass associated with ki	0.058 (18)	0.079 (17)	0.231 (26)	0.274 (11)
**k_m (1/day)**	Intermediate release rate constant	0.178 (34)	0.0643 (27)	0.122 (8)	0.0346 (64)
**f_m**	Fraction of the released drug mass associated with km	0.092 (18)	0.315 (16)	0.413 (15)	0.154 (38)
**Tdm (day)**	Mean transit time for drug release associated with km	1.5[b]	1[b]	3.7 (29)	11.8 (24)
**N_m**	Number of transit compartment for drug release associated with km	5[b]	5[b]	10[b]	10[b]
**k_s (1/day)**	Sustained release rate constant	0.00744 (16)	0.128	0.0134 (28)	0.0181 (16)
**Tds (day)**	Mean transit time for drug release associated with ks	18.1 (23)	50.8[b]	68.3 (7)	70.1 (9)
**N_s**	Number of transit compartment for drug release associated with ks	5[b]	6[b]	15[b]	15[b]
**F_{total}**	Projected total bioavailability of ISCD	1[b]	0.86 (7)	0.38 (7)	1[b]
**F_{tlast}**	Fraction of the total drug mass until the last observed concentration	0.80	0.86	0.35	0.97

[a] Coefficient of Variability (CV)%; [b] Fixed parameters

Table S3. The disposition PK parameter values obtained from the concentration-time profiles of TAC (0.075 mg/kg/day) and NAL (1 mg) following oral or IV administration in humans.

**Parameter (unit)**	**TAC**	**NAL**
**CL (L/hr)**	3.3	248
**V_{ss} (L)**	101	563
**k_{12} (1/hr)**	0.276	3.1
**k_{21} (1/hr)**	0.131	1.84
**$t_{1/2}$ (hr)**	24.8	1.83

Table S4. HPLC method details for different therapeutics

Therapeutic compound	Flow rate (mL/min)	Retention time (min)	Mobile phase	Gradient elution	Detection wavelength (nm)	Injection volume (μL)
TAC	1	19	methanol/DI water (70:30 v/v)	Table S5	210	20
LAM, ABC, NAL	0.45	13	10mM ammonium formate buffer/ACN (95:5 v/v)	Table S6	220-NAL<br>259-ABC<br>271-LAM	5
AMX	1	8	25 mM phosphate buffer/ACN (95:5 v/v)	None	240	20
VAN	1	8	20mM ammounium acetate buffer/methanol (88:12 v/v)	None	240	20

Table S5. Gradient program of the mobile phase for HPLC analysis of TAC

Time (min)	Mobile Phase	
	Methanol (%)	Water (%)
3	70	30
15	90	10
16	90	10
16.1	70	30
19	70	30

Table S6. Gradient program of the mobile phase for HPLC analysis of LAM, ABA, and NAL

Time (min)	Mobile Phase	
	Methanol (%)	Water (%)
2	95	5
3	95	5
7	40	60
8	40	60
8.1	95	5
3	95	5
